# A Programmable Multi‑Modal Information Encryption System Based on the Dynamic Wetting Behavior of Liquid Metal

**DOI:** 10.1002/advs.75745

**Published:** 2026-06-26

**Authors:** Xuedong Qiang, Min Mao, Yihao Wang, Nan Li, Mengyu Jiang, Chenkun Sun, Bangdeng Du, Jifei Ye, Linyan Wang, Fei Zhan, Lei Wang

**Affiliations:** ^1^ Nantong Institute of Metrology & Measurement Jiangsu China; ^2^ State Key Laboratory of Efficient Production of Forest Resources, Beijing Key Laboratory of Lignocellulosic Chemistry Beijing Forestry University Beijing China; ^3^ Beijing Key Laboratory of Cryo‐Biomedical Engineering, Technical Institute of Physics and Chemistry Chinese Academy of Sciences Beijing China; ^4^ State Key Laboratory of Lunar and Planetary Sciences Macau University of Science and Technology Macau China; ^5^ State Key Laboratory of Advanced Space Propulsion Department of Aerospace Science and Technology Space Engineering University Beijing China

**Keywords:** dynamic wetting, image, Information encryption, liquid metal, textual

## Abstract

Information encryption and security are the cornerstones of the digital era. However, traditional optical and electronic encryption techniques remain vulnerable to decryption due to deterministic material responses and predictable algorithms. In this study, we present a programmable multi‑modal information encryption system based on the dynamic wetting behavior of liquid metal. The system integrates an inclinometer sensor that utilizes the dynamic wetting characteristics of liquid metal, a probe‑driven transmission mechanism, and a three‑dimensional motion platform. Through the coupling of mechanical regulation and fluid dynamics, the system achieves secure physical information encoding with multidimensional and reconfigurable encryption capability. Proof‑of‑concept experiments demonstrate that the system exhibits high precision and stability in the encryption and decryption of image and textual information. The proposed framework employs four independent encryption parameters, and successful decoding requires deviations smaller than 0.02%. Overall, this research establishes a controllable and highly secure materials‑driven encryption framework, providing a promising route for advanced information protection and encryption technologies.

## Graphical abstract

This work presents a programmable multimodal information encryption device that harnesses the dynamic wetting behavior of liquid metal to achieve secure, reconfigurable, and high‐precision encryption and decryption of diverse data types, including text and images. The system demonstrates enhanced multimodal integration, stability, and adaptability for advanced information protection.

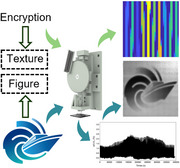

## 1 Introduction

Information security is a cornerstone of the digital era, encompassing the protection of individual and corporate interests while safeguarding national security and social stability [1, 2, 3, 4]. Throughout history, societies have continuously developed innovative methods to prevent unauthorized access to sensitive information. With rapid advances in network technologies and the exponential growth of data exchange, encryption has become indispensable for authentication and secure transmission, serving as a primary defense against the forgery and tampering of personal information and intellectual property [5, 6]. Encrypted information generally falls into two categories, which are textual data (e.g., documents, messages, identity credentials) and image data (e.g., digital images, watermarks, visual identity verification). Due to their distinct characteristics, targeted encryption schemes are required for each type. Various security technologies have thus been developed, including radio‐frequency identification (RFID) tags [7], fluorescent and phosphorescent materials [8, 9, 10], invisible inks [11], holograms [12] , watermarks [13], and cryptographic methods [14, 15, 16], providing multi‐layered solutions across different application scenarios. Nevertheless, current encryption technologies still face significant limitations. Optical encryption methods, which often employ fluorescent or phosphorescent materials, rely heavily on luminescent characteristics such as photoluminescence intensity, emission wavelength, and lifetime behavior to secure image‐based data. However, once the material composition or luminescence mechanism becomes known, security can be compromised [17, 18, 19]. Likewise, textual encryption methods, including classical cryptographic systems such as the Caesar cipher, the scytale device, and coded telegraphs, remain vulnerable to modern cryptanalysis, which exploits statistical features of encoded symbols to infer the original information [20].

To overcome these limitations, Physical Unclonable Functions (PUFs) have emerged as a promising alternative [21, 22, 23]. PUFs generate unique and irreproducible cryptographic keys by utilizing random physical variations introduced during material fabrication, offering distinct advantages for anti‐counterfeiting and secure authentication   [24]. Since the concept was first proposed by Pappu et al. [25], multiple PUF mechanisms have been explored, including optical [26, 27, 28], magnetic [29, 30, 31] , and electronic implementations [32, 33]. Among these, optical PUFs are particularly attractive due to their high data encoding capacity, complex output responses, and broad potential applications driven by inherent material randomness [34]. However, conventional PUFs often exhibit fixed and non‐reconfigurable key generation with limited controllability, rendering them unsuitable for long‐term or renewable credentials [35, 36, 37, 38, 39].

This study proposes a programmable multi‑modal information encryption system based on the dynamic wetting behavior of liquid metal (LM). The system architecture consists of three core modules, including an inclinometer sensor that utilizes the dynamic wetting behavior of LM, a transmission mechanism driven by a precision‑controlled probe, and a three‑dimensional motion platform. During operation, the three‑dimensional motion platform precisely controls the probe's scanning trajectory to perform point‑by‑point scanning of molds that contain image or textual information. The inclinometer sensor generates corresponding tilt responses according to variations in surface height and converts them into electrical signals, thereby establishing a mapping relationship between the signals and the surface morphology. The system achieves multi‑modal encryption through interference between different types of information. By combining adjustable scanning parameters with programmable control of the three‑dimensional motion platform, multiple encryption dimensions are introduced [38, 39]. Proof‑of‑concept experimental results demonstrate that the proposed system can accurately encrypt and decrypt both corporate logo patterns and textual information. It is worth noting that the system employs four independent encryption parameters, and successful decryption requires parameter deviations of less than 0.02%. Overall, the encryption framework developed in this study exhibits a high level of controllability and strong multi‑modal encoding capability, providing a feasible and efficient approach for materials‑based physical information protection.

## 2 Results and Discussion

Over billions of years of environmental change, organisms in nature have continuously evolved and diversified. The unique natural structures and operating mechanisms that have emerged during these processes have consistently inspired scientific and technological progress, enabling researchers to establish biomimetic design strategies. Specifically, plant surfaces have evolved microscale morphological structures with remarkable diversity and precision, displaying a level of refinement comparable to works of art and exceeding current fabrication capabilities. Thus, using natural plant structures as templates provides an effective approach to overcoming the limitations of existing technologies in specific research areas [40].

**FIGURE 1:**
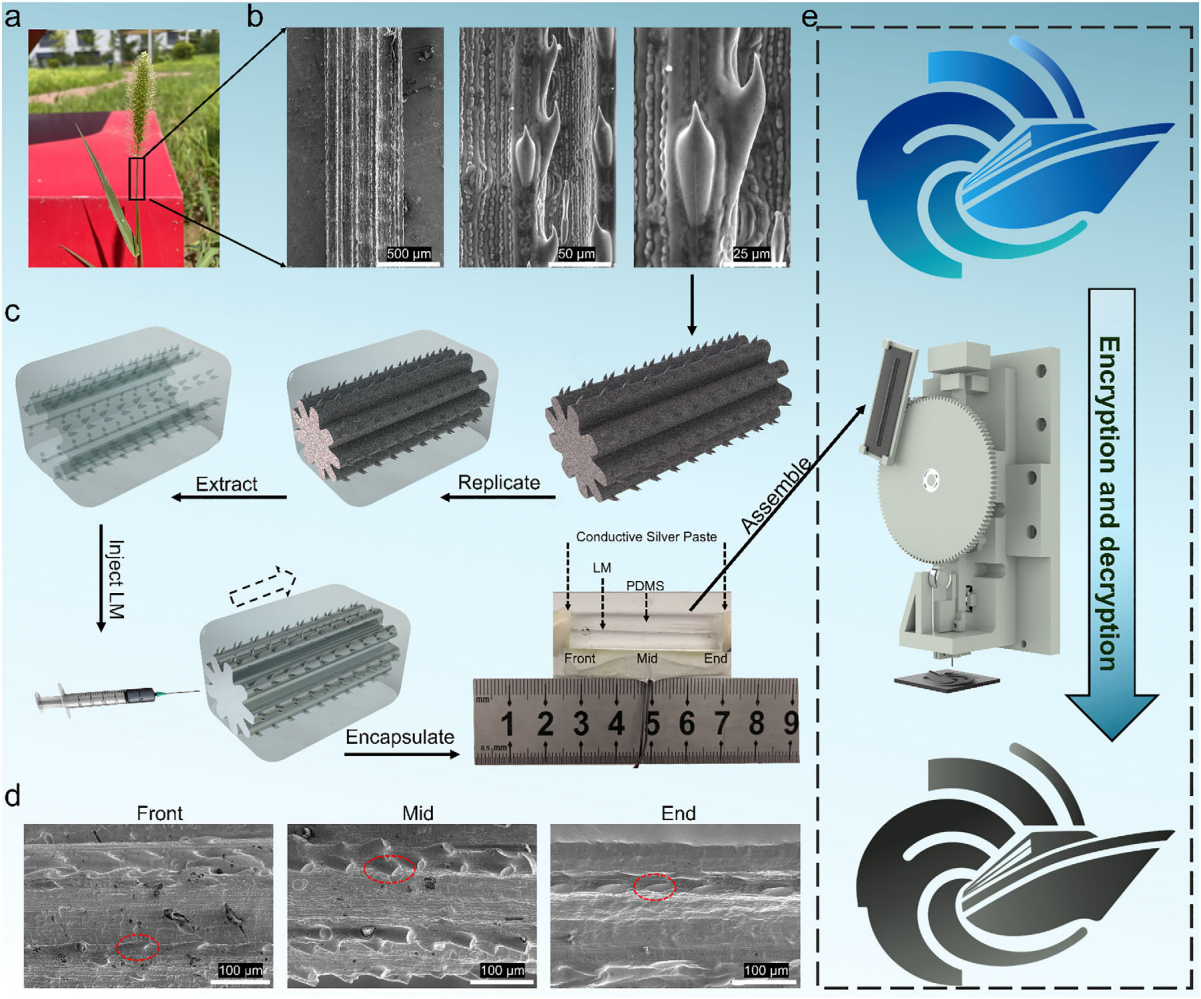
Fabrication and application of inclinometer sensor. (a) Natural state of Setaria viridis. (b) SEM characterization of the surface morphology of the stem of Setaria viridis. (c) Fabrication of inclinometer sensor. (d) Gradient distribution of LM inside the microchannel. (e) The combination of the inclinometer sensor with transmission components and a 3D motion platform enables multimodal information encryption.

In this study, Setaria viridis was used as a natural template. The stem of Setaria viridis is densely covered with obliquely oriented conical microstructures, each with an average size of approximately 25 µm (Figure 1a,b). Polydimethylsiloxane (PDMS) was employed to replicate these microstructures on the stem surface. The stem was immersed in PDMS prepolymer, followed by vacuum degassing and thermal curing to ensure complete infiltration. After curing, the Setaria viridis stem was carefully removed, yielding a microchannel whose inner wall was patterned with micro‑conical, pit‑like structures. LM was then injected into the microchannel using a syringe, and both ends were sealed and equipped with electrodes using conductive silver paste. Through this process, an inclinometer sensor based on the dynamic wetting behavior of LM was successfully fabricated (Figure 1c). In addition, to further examine the distribution of LM within the microchannel, a high‑melting‑point alloy was injected as a substitute for the gallium‑based LM under a high‑temperature environment of 80°C. After cooling and solidification, the alloy sample was carefully removed, allowing direct visualization of the distribution state of the gallium‑based LM inside the microchannel (Figure 1d). SEM observations revealed that the LM exhibited distinct filling behaviors along the microchannel. Near the injection port, the LM completely occupied the microstructures, whereas at positions farther from the inlet, only a small fraction of the microcavities was filled. According to the extent to which the LM is pressed into the micro‑pits, it can be roughly divided into three sections, namely the front, middle, and end regions. It is expected that by integrating the inclinometer sensor with a 3D motion platform and mechanical transmission components, multidimensional and multimodal information encryption can be achieved (Figure 1e).

**FIGURE 2:**
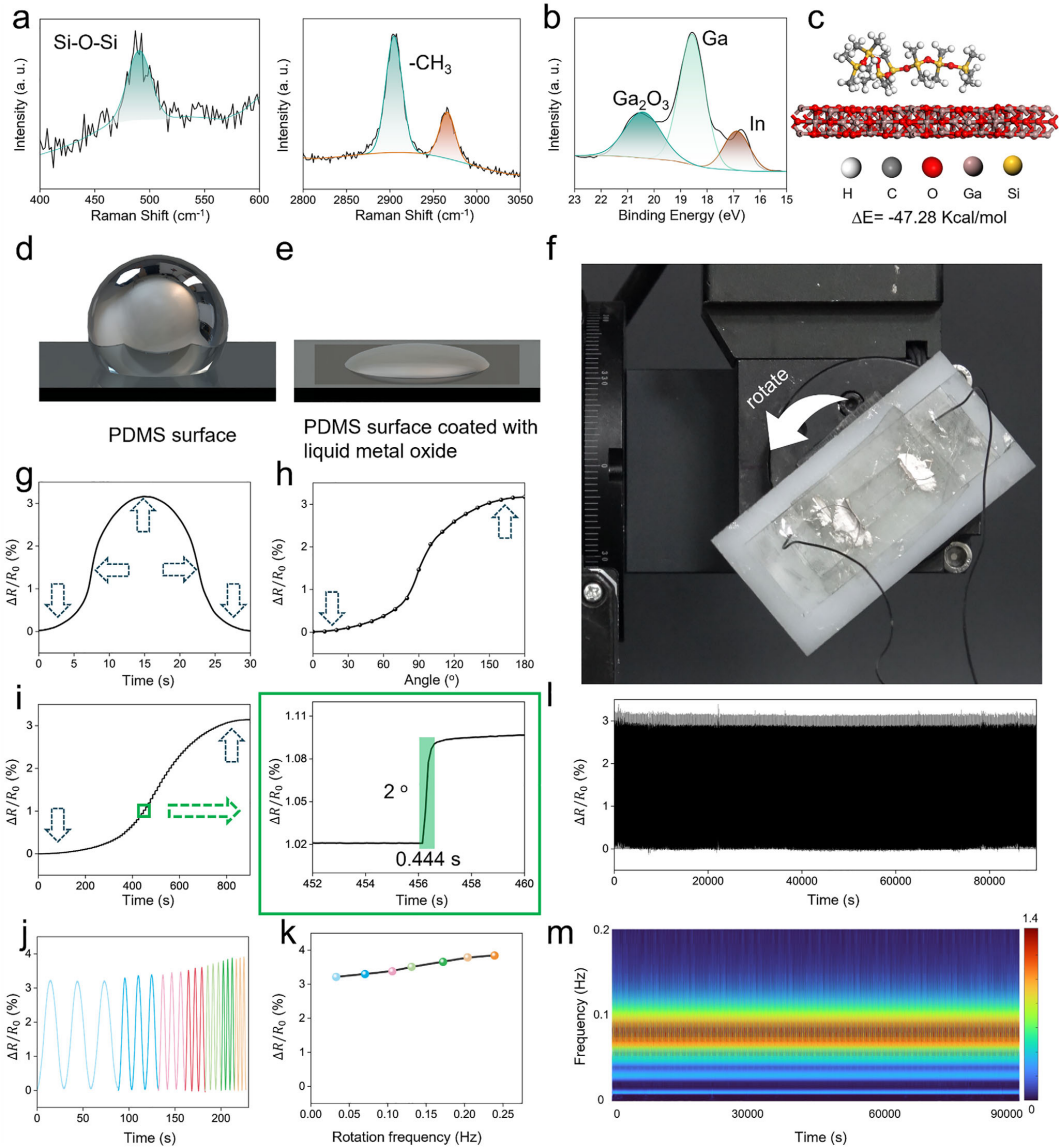
Interfacial adhesion and electrical characteristics of the inclinometer sensor. (a) Raman spectra of the PDMS. (b) XPS of LM. (c) Molecular dynamics simulation of the adhesion interaction between LM oxide and PDMS. (d,e) Sketches of differential wetting characteristics of LM on PDMS surface and its oxide layer. (f) Schematic diagram of the tilt angle signal testing process. (g) Rate of electrical signal change generated by one full rotation of the inclination sensor. (h) Rate of electrical signal change at different tilt angles. (i) Accuracy and response time of inclination sensor. (j,k) Change in amplitude of periodic electrical signals during a single cycle of increase in rotational frequency from 0.03 to 0.25 Hz. (l,m) Durability testing of the inclination sensor and wavelet transform data.

We further investigated the interfacial adhesion mechanism between the oxide layer of the LM and the PDMS substrate. The strong interfacial adhesion effectively anchors the LM within the microchannel and maintains the long‑term stability of the solid–liquid–gas three‑phase interface. This characteristic is expected to enhance the long‑term operational stability of the inclinometer sensor based on the dynamic wetting behavior of LM. Specifically, the Raman spectrum of PDMS confirmed the presence of Si─O─Si bonds and ─CH_3_ groups on its surface (Figure 2a). Meanwhile, X‑ray photoelectron spectroscopy (XPS) provided evidence for the formation of an oxide layer on the LM surface (Figure 2b). The Ga 2p spectrum further revealed that exposure to air induces the oxidation of LM to form a Ga_2_O_3_ layer, corresponding to the Ga (III) peak at a binding energy of 1118.1 eV. By integrating these experimental results with molecular dynamics simulation, the interfacial adhesion between PDMS and LM can be attributed mainly to van der Waals forces, which arise from interactions between Ga/O atoms on the LM surface and Si/O/C/H atoms on the PDMS surface. The calculated interfacial binding energy between a single PDMS molecular chain and the LM oxide layer is −47.28 kcal mol^−1^ (Figure 2c). Although such forces are intrinsically weak, the LM oxide layer behaves as a “soft solid,” enabling intimate conformal contact with the PDMS surface. As a result, the extensive contact area generates a cumulative effect that magnifies the overall van der Waals interaction, leading to strong macroscopic adhesion at the LM‐PDMS interface. Moreover, the wettability of the LM on the PDMS surface differs markedly from that on the PDMS surface coated with an oxide layer (Figure 2d,e). Such a difference enables the LM at the interface to stay in a metastable state, thereby exhibiting dynamic wetting behavior in response to varying additional pressure. In this study, gravitational force is utilized as the source of external pressure for the inclinometer sensor. By varying the tilt angle, the magnitude of the applied pressure acting on the LM can be precisely tuned, allowing controllable dynamic wetting. Consequently, the deformation arising from the dynamic wetting of the LM induces corresponding changes in electrical resistance, which could be detected as the sensor's output signals.

During the electrical characterization of the inclinometer sensor, the tilt angle was precisely regulated by a motorized rotation stage, ensuring high accuracy and excellent repeatability of angular adjustment throughout all experiments. Meanwhile, an Agilent 34420A high‑precision multimeter was used to continuously monitor and record the real time resistance variations of the sensor, enabling high resolution electrical data acquisition. At the start of each measurement cycle, the sensor was positioned vertically downward (Figure 2f) and subsequently rotated clockwise in a programmable manner by the motorized stage. This setup provided well‑controlled experimental conditions for a systematic and comprehensive evaluation of the sensor's electromechanical response to angular displacement.

When rotated clockwise at a constant angular velocity through a complete 360°, the sensor exhibited a stable and well‑defined sinusoidal variation in electrical signal with respect to the tilt angle (Figure 2g). As the inclination increased from 0° to 180°, the signal variation followed a typical sinusoidal trend with a maximum amplitude of 3% (Figure 2h). Such periodic behavior clearly evidences a robust and repeatable electromechanical coupling between angular displacement and electrical output. The sensor further demonstrated a high angular resolution, capable of discerning minute variations down to 2° (Figure 2i). Its output signals exhibited high precision, which verifying reliable performance under static conditions. Beyond the remarkable static performance, the sensor also displayed superior dynamic responsiveness. Under rapidly varying tilt angles, it produced fast and accurate responses, with step‑like resistance transitions completed within 0.44 s. This rapid response underscores the sensor's exceptional sensitivity and mechanical robustness, enabling real‑time tracking and precise detection of angular variations. The long‑term operational stability of the sensor was further validated through durability testing. During continuous operation exceeding 90 000 s, the electrical signal remained remarkably stable without discernible drift, demonstrating excellent temporal stability and reliability (Figure 2l). Moreover, wavelet transform analysis of the recorded signals revealed highly consistent and reproducible spectral features, further confirming the superior robustness and signal fidelity of the sensor under prolonged continuous operation and high‑frequency measurement conditions (Figure 2m). Collectively, these characteristics demonstrate the strong potential of the device for dynamic sensing.

**FIGURE 3:**
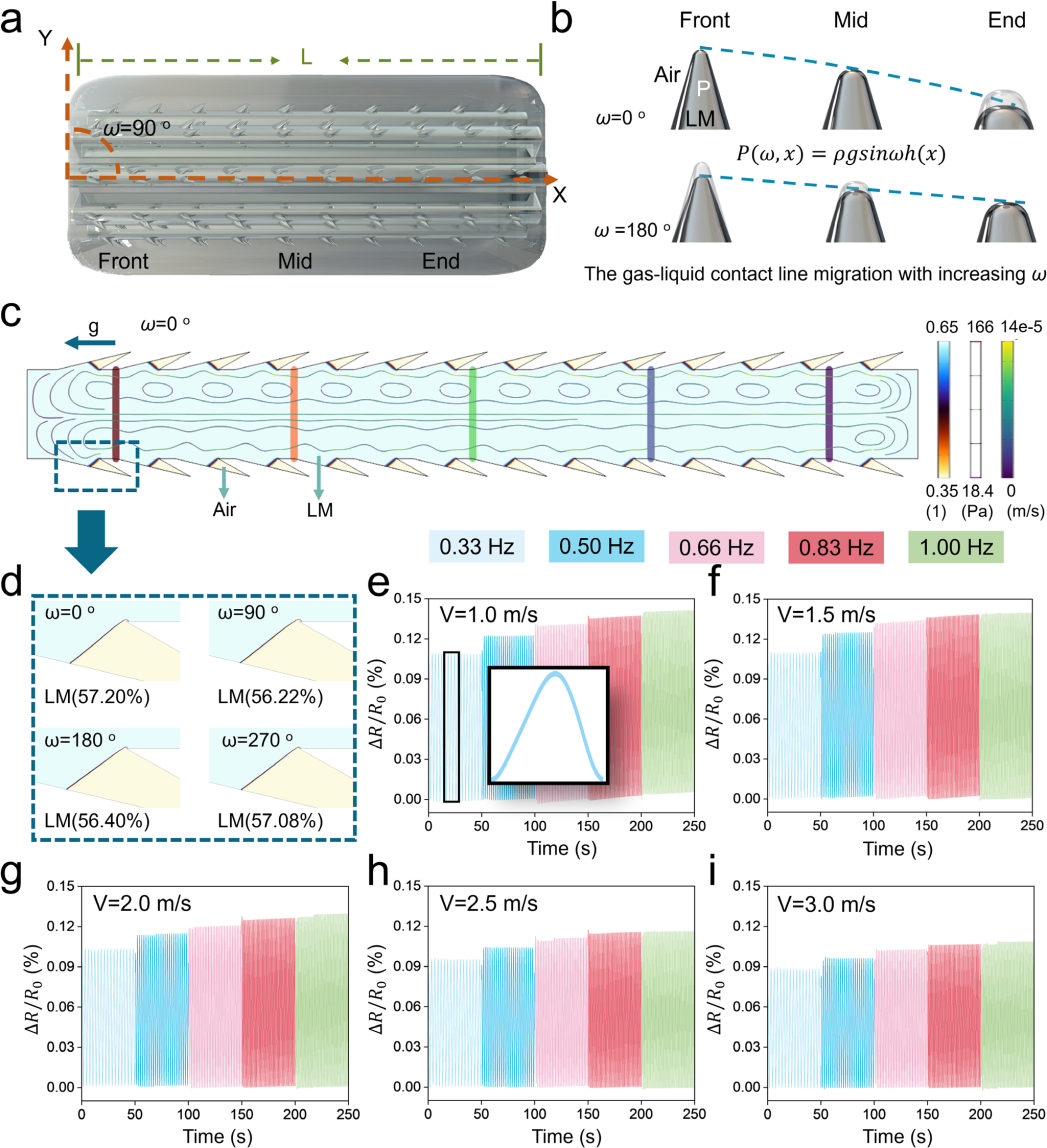
Working mechanism and simulation of the inclinometer sensor. (a) Schematic diagram of the gradient distribution of LM inside the microchannels. (b) Schematic diagram of the three‐phase interface migration. (c) Phase‐field simulation of the inclinometer sensor. (d) Simulation of the deformation of LM within the microstructures under additional pressure. (e–i) Simulation of the effects of the injection velocity of the LM and the rotation speed of the inclinometer sensor on the characteristics of the electrical signal response.

The observed sinusoidal variation of the resistance signal with changes in tilt angle may be attributed to gravitationally induced additional pressure acting on the LM within the microstructures, in conjunction with its gradient distribution inside the microchannels. On the one hand, the additional pressure exerted on the LM within the microstructures of the inclinometer sensor is directly associated with the static pressure in the microchannels. For the LM located at a distance *x* from the injection outlet, the static pressure is determined by the relative height at that position, which varies sinusoidally as the tilt angle increases. As a result, the static pressure of the LM exhibits a distinct sinusoidal dependence on the tilt angle (Figure 3a). And when the tilt angle of the inclinometer sensor is *ω*, the static pressure of the LM located at a distance *x* from the injection port can be expressed as [41]:

(1) P(ω,x)=ρgsinωh(x)

On the other hand, the LM forms a gradient distribution during the injection process due to the pressure drop, resulting in an overall nonuniform configuration within the microchannels [42]. Taking the initial horizontal state of the inclinometer sensor as an example, when the sensor is tilted clockwise, the region of the LM with a larger cross‑sectional area shifts upward, whereas the region with a smaller cross‑sectional area moves downward. This positional redistribution causes the lower region with a smaller cross‑sectional area to experience higher additional pressure, thereby promoting an expansion of its cross‑section, while the upper region correspondingly shrinks [43]. Conversely, when the sensor is tilted counterclockwise, the trend of variation reverses. Such periodic deformation of the LM with respect to the tilt angle directly gives rise to the corresponding resistance response of the sensor. And the overall behavior manifests as a periodic migration of the solid–liquid–gas three‑phase interface (Figure 3b).

To validate the deformation behavior of the LM within the microstructures under additional pressure, simulations were conducted. Specifically, starting from the initial vertically downward orientation, the LM content in the gap decreases from 57.2% to 56.2% as the inclinometer sensor rotates clockwise from 0° to 90°. With continued clockwise rotation to 180°, the LM content begins to increase, reaching 56.4%. As the rotation angle further increases from 180° to 270°, the LM content continues to rise, reaching 57.08% (Figure 3c,d). In addition, a comprehensive simulation analysis was performed to examine the influence of LM injection velocity and tilt sensor rotation speed on the electrical signal characteristics. The results revealed that as the injection velocity increased from 1 to 3 m s^−1^, the rate of change in the tilt sensor's electrical signal exhibited a slight decrease, indicating that higher injection velocities tend to diminish the sensor's responsiveness. In contrast, when the rotation speed was raised from 1/3 to 1 Hz, the signal variation rate progressively increased, suggesting that enhanced rotational motion strengthens the electrical output intensity (Figure 3e–i). These trends can be attributed to the distinct flow and deformation behaviors of the LM under varying dynamic conditions [44]. Increasing the injection velocity elevates the dynamic pressure, causing the LM to penetrate more deeply into the microscopic pores and consequently constraining its effective deformation range, which leads to a reduced rate of electrical signal variation. Conversely, an elevated rotational speed generates additional pressure through centrifugal effects, intensifying the strain response at the LM interface and thereby enhancing the signal variation rate. Furthermore, the simulation results demonstrate a sinusoidal relationship between the electrical signal and the tilt angle, indicating that the sensor's electrical response exhibits a predictable and periodic dependence on angular position. Overall, the simulated results show excellent agreement with experimental observations, thereby reinforcing the reliability and accuracy of the proposed theoretical model. This strong consistency underscores the robust performance of the sensor under complex dynamic conditions and provides important insights for optimizing its operational parameters to improve measurement precision and efficiency.

**FIGURE 4:**
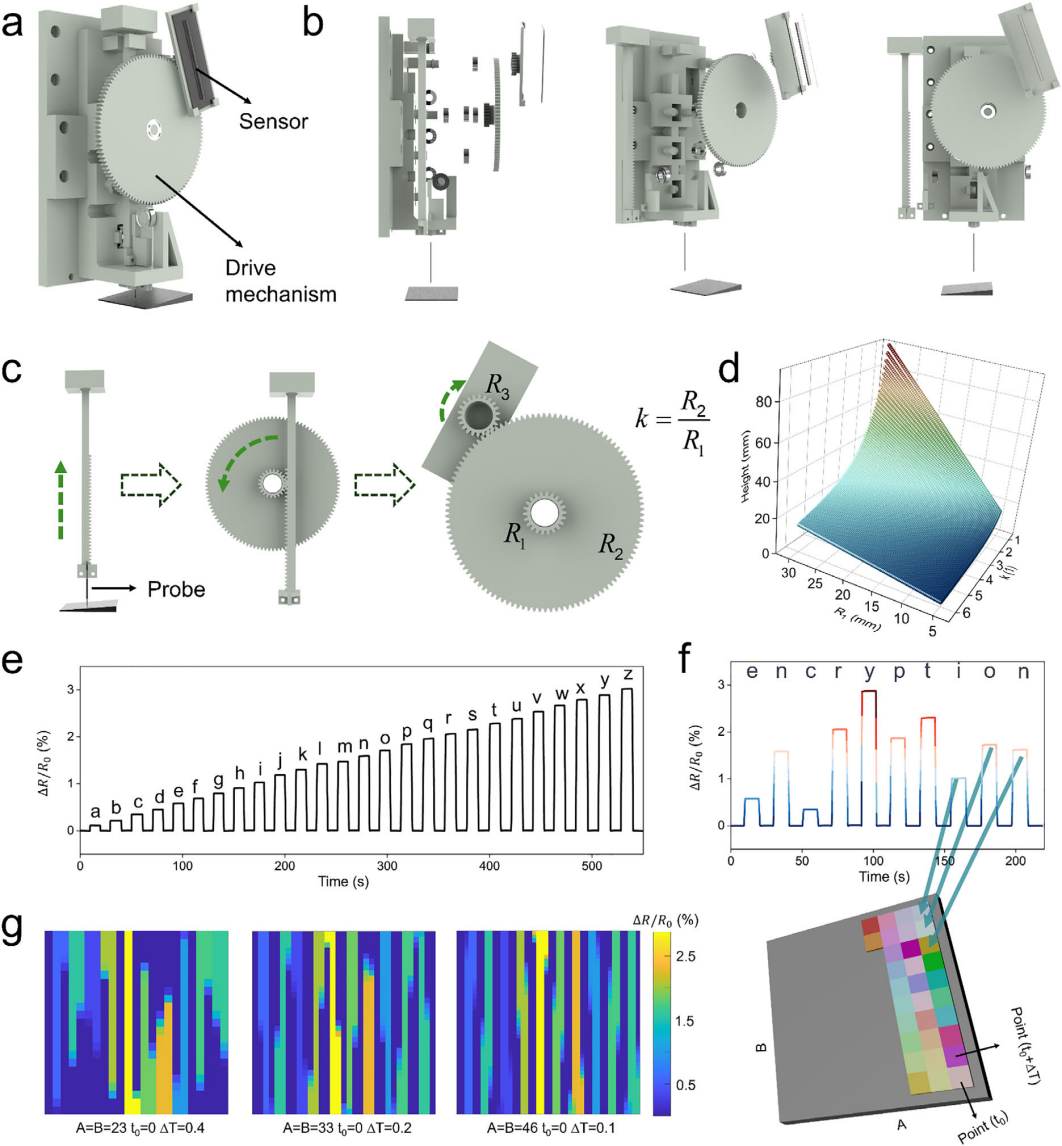
Assembly of encryption devices and text encryption. (a) Schematic diagram of the encryption device. (b) Exploded view of the encryption device. (c) Schematic illustration of the mechanical transmission between the probe and the inclinometer sensor. (d) Relationship between the probe's detection height and the gear design parameters. (e) Calibration of textual characters using the peak rate of change in electrical signals. (f) The textual data “encryption” is converted into electrical signal form using the encryption device and subsequently further encoded into an image representation. (g) Image representations obtained under different encryption parameters.

In addition, by integrating the inclinometer sensor with the transmission device, coordinated operation was achieved under the control of the three‑dimensional motion platform, forming a multimodal signal‑response system based on the dynamic wetting behavior of LM. This configuration fully exploits the tunable wetting characteristics of the liquid‑metal interface under mechanical perturbation, enabling a programmable coupling between mechanical input and electrical output. The resulting physical encryption mechanism realizes dynamic mapping and re‑encoding of multimodal information, highlighting the unique advantages of LM in reconfigurable physical information processing. (Figure 4a). Customized structural components were fabricated by 3D printing and securely integrated into a 3D motion platform, with the inclinometer sensor precisely fitted into the slot at its top [45]. The encryption module comprises a probe coupled to a rack‑and‑pinion assembly, where the rack engages gear R_1_. Gear R_1_ is mechanically linked to rotary disk R_2_, which further meshes with gear R_3_ located behind the slot (Figure 4a). During encryption, the probe descends in discrete steps to contact the gradient surface [46, 47]. The subsequent upward motion of the rack drives gear R_1_ to rotate counterclockwise, which is transmitted through rotary disk R_2_ to gear R_3_, inducing a clockwise rotation that yields a tilt signal corresponding to the probe's displacement (Figure 4b,c). Simultaneously, the inclinometer sensor exhibits a high‑precision electrical response. Under programmed control of the motion platform, the probe establishes deterministic mechanical contact with the gradient surface, producing electrical signals characterized by distinct peaks that encode different textual symbols.

Benefiting from the inclinometer sensor's high resolution, rapid response, long‑term stability, and its unique sensing mechanism based on the dynamic wetting behavior of LM, the encryption system achieves high‑precision encoding of textual data. To further enhance system performance, the diameters of gears R_1_, R_2_, and R_3_, as well as the surface gradient configuration, were precisely optimized to match the inclinometer sensor's maximum detection range of 180°, thereby ensuring the reconfigurability and controllability of the encryption process. This design consideration maximizes the differentiation of electrical signal peaks corresponding to distinct textual characters and enhances the accuracy of the encryption process. For clarity of presentation, the relationship among the diameters of the three gears is formulated as *R*_1_ = *R*_3_, $k=\frac{R_{2}}{R_{1}}$. And given the limited detection range of the inclinometer sensor, the maximum height variation of the gradient surface employed for calibrating the letter information is described as [48, 49]:

(2) $$H=\frac{\pi R_{1}}{k}$$

With a rational structural design, the maximum height difference can reach up to 90 mm, which facilitates easier calibration of different letters (Figure 4d). In the experimental setup, gears R_1_ and R_2_ were fabricated with diameters of 16 mm and 110 mm, respectively [50]. This configuration allowed precise calibration of distinct letter patterns. Under the programmed control of the 3D motion platform, the word “encryption” was further encoded into a sequence of electrical signals (Figure 4e,f). In the subsequent processing stage, the acquired electrical signal data were retrieved, extracted, and reconstructed in the MATLAB environment, with signal amplitudes mapped to corresponding colors. This procedure enabled the transformation of the electrical signals into image form, while the embedded textual information was subjected to further encryption processing (Figure 4g). Specifically, parameters A and B were used to define the dimensions of the encrypted image, while parameter x determined the temporal position of the first extracted point, and parameter y controlled the time interval between adjacent extracted points. Based on these multidimensional parameters, the information was encrypted and mapped into three distinct image forms. The original information could only be accurately decoded when all parameters were correctly specified.

In addition to converting and encrypting textual information into image form through electrical signals, we can also directly encrypt image data into electrical signal form. However, compared with the encryption process for textual information, this procedure is relatively more complex in practice.

**FIGURE 5:**
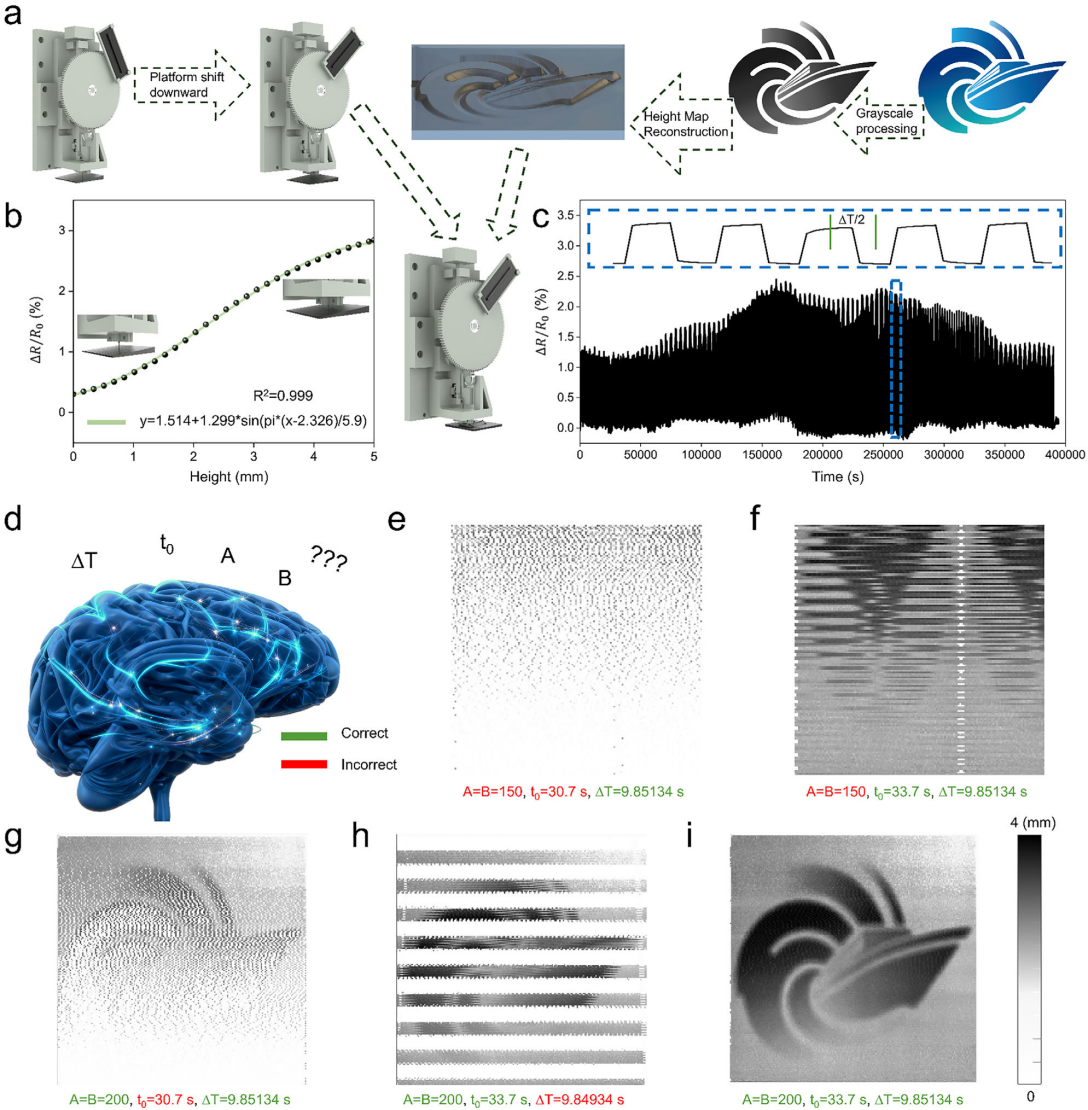
The image information was encrypted using the encryption device. (a) Process of image information encryption. (b) Response relationship between probe lift height and electrical‑signal variation rate. (c) Electrical signal data containing hidden image information. (d) Parameters required for the decoding of electrical signal data. (e–i) The effect of parameter errors on the decoding of electrical signal data.

Specifically, the image to be encrypted was first converted into a grayscale representation according to its color distribution and brightness variations. This conversion eliminates the interference of color dimensions and highlights the structural and hierarchical characteristics of the image, thereby enabling precise extraction of grayscale‑level information. This step provides the fundamental data foundation for subsequent spatial modeling. Subsequently, the displacement‑mapping function in 3D Studio Max, based on grayscale values, was used to map the grayscale data into height information corresponding to the brightness depth. Through this mapping process, the two‑dimensional brightness variations of the original image were transformed into spatial height fluctuations, achieving the mapping and reconstruction from planar grayscale distribution to 3D structural morphology. The resulting 3D morphological model not only accurately reflects the brightness variation patterns of the original image in geometric form but also serves as a precise spatial carrier for subsequent physical printing and encrypted signal generation (Figure 5a). After the construction and printing of the 3D morphological model, a systematic calibration was performed to further achieve precise control over the encryption and interpretation accuracy at the electrical‑signal level. In this process, the relationship between the probe's lift height and the rate of change of the electrical signal detected by the inclinometer sensor was calibrated through multiple repeated measurements conducted under various displacement increments and scanning speeds, thereby establishing a quantitative correlation model between mechanical displacement and electrical‑signal output (Figure 5b). This mode not only elucidates the dynamic coupling behavior between probe displacement and signal response but also provides both theoretical and experimental foundations for the accurate interpretation and error correction of subsequent electrical‑signal data. On the basis of the established calibration, the logo of the Nantong Institute of Metrology & Measurement was subsequently employed as a representative sample to demonstrate the proposed encryption process (Figure 5c). In the subsequent experiments, four critical parameters, A, B, T_0_, and ∆T, were utilized to systematically decode the electrical signal data produced by the encryption device after the image information had been encrypted (Figure 5d). The results reveal that accurate reconstruction of the original image information can be achieved only when the relative errors of all four parameters are maintained below 0.02%. Once any single parameter exceeds this threshold, noticeable distortion appears in the decoded image (Figure 5e–i). These findings indicate that the proposed encryption and decryption approach exhibits extremely high sensitivity to parameter accuracy, thereby validating both the precision and the security of the developed encryption mechanism.

## 3 Discussion

In summary, this study presents a multidimensional encryption strategy based on a mechanically tunable PUF. The system integrates a LM inclinometer sensor, transmission mechanism, and 3D motion platform. By utilizing the dynamic wetting behavior of LM within biomimetically replicated microchannels, the fabricated inclinometer sensor exhibits high sensitivity, excellent stability, and a predictable coupling between the tilt angle and electrical resistance. Experimental characterization and numerical simulations reveal that the sinusoidal electrical response of the sensor originates from gravitationally induced pressure variations and the gradient distribution of LM inside the microchannels, thereby enabling highly precise and repeatable angle detection. Integration of the inclinometer sensor with the mechanically driven transmission module enables multimodal information encryption, allowing textual and image data to be converted into distinctive electrical signal patterns. The system employs four independent encryption parameters, and effective decryption can be achieved only when the relative error of all parameters is less than 0.02%, demonstrating the innovation and feasibility of this approach in information mapping and encryption mechanisms. This study constructs a novel mechanically controllable and reconfigurable physical encryption system that exhibits significant application potential in the field of information encryption and security. By establishing a synergistic mechanism among mechanical regulation, material properties, and signal response, the system not only enhances the controllability and reliability of the encryption process but also provides new research perspectives and a technical framework for the development of related technologies such as anti‑counterfeiting and intelligent sensing.

Nevertheless, certain limitations remain. When the system is employed for high‑resolution encryption of textual or image information, the overall encryption cycle tends to be relatively long. This limitation primarily arises from the current control configuration of the 3D motion platform. In future work, the efficiency of the encryption process can be further improved by adopting motion actuators with faster response or by optimizing the structural design and control algorithms of the transmission mechanism, thereby enhancing the system's overall performance and practicality.

## 4 Methods

### 4.1 Preparation of Inclinometer Sensor

First, the surface morphology of Setaria viridis was replicated using PDMS, with a mass ratio of 10:1 between the PDMS base and the curing agent. After the Setaria viridis specimen was immersed in liquid PDMS, the assembly was placed in a vacuum oven and degassed at −0.08 MPa for 0.5 h to eliminate trapped air bubbles. The pressure was subsequently restored to the atmospheric level, and the sample was thermally cured at 65°C for 2 h to ensure complete polymerization of the PDMS. Upon curing, the Setaria viridis template was carefully removed, yielding a microchannel structure with finely microstructured inner walls. For the fabrication of the inclinometer sensor, LM was injected into the prepared microchannels at a speed of 1 m·s^−^^1^, with the injection rate precisely controlled by a peristaltic pump. After the channels were fully filled, both ends were sealed with conductive silver paste, and electrodes were formed by attaching conductive wires. Finally, the device was placed in a vacuum oven and maintained at 65°C for 1 h to sinter and cure the silver paste, thereby completing the sensor fabrication.

### 4.2 Design of the Transmission Mechanism

All transmission components were modeled and designed using SolidWorks 2021, and fabricated through 3D printing technology.

### 4.3 Record of Electrical Signals

The electrical signals generated during the rotational movement of the sample were meticulously measured using a high‐precision digital multimeter (Agilent 34420A, USA). Meanwhile, the data were collected using the accompanying software of the device, BenchVue Basic Digital Multimeter App (2019 Update 4.0).

### 4.4 Raman Analysis of PDMS

The Si─O─Si and ─CH_3_ groups on the surface of PDMS were determined by confocal Raman microscope (inVia‐Qontor).

### 4.5 XPS Analysis of LM

The Ga_2_O_3_ of LM surface was determined by X‐Ray Photoelectron Spectroscopy (ESCALAB250 Xi).

### 4.6 SEM Characterization

The sample was mounted on the stage with conductive adhesive and gold‐sputtered for 60 s prior to testing. Imaging was performed on a Hitachi SU8600 field‐emission scanning electron microscope in secondary‐electron mode at an accelerating voltage of 3 kV.

### 4.7 Numerical Simulation

Numerical theory simulations were performed using the commercial software COMSOL Multiphysics (Version 5.6, COMSOL Inc., Sweden). Specifically, the simulation involves the coupling of multiple physical fields, including laminar flow, phase field, and electric current field, in which the laminar flow and phase field are coupled through the phase‑field interaction method. The model considers two materials, LM and air. The parameters for air are adopted directly from the default values in the COMSOL built‑in material library, while the parameters for LM are set as a density of 6280 kg/m^3^, a dynamic viscosity of 0.00322 Pa·s, and a relative permittivity of 15. The mesh type is physics‑controlled, and the element size is refined. For the laminar‑flow field, a parametric scan of the inlet injection velocity was performed with values of 1, 1.5, 2, 2.5, and 3 m/s. At the same time, a parametric scan of the rotational speed in the direction of gravity was carried out with all combinations of 0.33, 0.5, 0.66, 0.83, and 1 Hz. The transient solution consists of two consecutive steps. The first step is the injection process of LM with a time step of 0.01 s, and the solution from the final time step is used as the initial condition for the next step. The second step is the rotation process with a time step of 0.1 s. All related parameter definitions, boundary conditions, and solver settings have been described in detail in the revised manuscript to enable other researchers to accurately reproduce the results of this study using COMSOL Multiphysics.

## Author Contributions

L. W. and F. Z. conceptualized the idea and supervised the research. X. Q., M. M., Y. W., contributed equally to this work and considered to be the first co‐author. X. Q., M. M., Y. W., N. L., M. J., C. S., B. D., J. Y., and L. W., designed the experiment, collected the datasets. X. Q., M. M., Y. W and F. Z., wrote the manuscript. All the authors read, contributed to the discussion, and approved the final manuscript.

## Conflicts of Interest

The authors declare no conflicts of interest.

## Data Availability

The data that support the findings of this study are available from the corresponding author upon reasonable request.
